# Vascular smooth muscle contraction in hypertension

**DOI:** 10.1093/cvr/cvy023

**Published:** 2018-01-31

**Authors:** Rhian M Touyz, Rheure Alves-Lopes, Francisco J Rios, Livia L Camargo, Aikaterini Anagnostopoulou, Anders Arner, Augusto C Montezano

**Affiliations:** 1 BHF Glasgow Cardiovascular Research Centre, Institute of Cardiovascular and Medical Sciences, University of Glasgow, 126 University Place, Glasgow G12 8TA, UK; 2 Department of Physiology and Pharmacology, Karolinska Institutet, Stockholm, Sweden

**Keywords:** Contraction, Dilation, Calcium, Actin cytoskeleton, Rho kinase, Vascular tone

## Abstract

Hypertension is a major risk factor for many common chronic diseases, such as heart failure, myocardial infarction, stroke, vascular dementia, and chronic kidney disease. Pathophysiological mechanisms contributing to the development of hypertension include increased vascular resistance, determined in large part by reduced vascular diameter due to increased vascular contraction and arterial remodelling. These processes are regulated by complex-interacting systems such as the renin-angiotensin-aldosterone system, sympathetic nervous system, immune activation, and oxidative stress, which influence vascular smooth muscle function. Vascular smooth muscle cells are highly plastic and in pathological conditions undergo phenotypic changes from a contractile to a proliferative state. Vascular smooth muscle contraction is triggered by an increase in intracellular free calcium concentration ([Ca^2+^]_i_), promoting actin–myosin cross-bridge formation. Growing evidence indicates that contraction is also regulated by calcium-independent mechanisms involving RhoA-Rho kinase, protein Kinase C and mitogen-activated protein kinase signalling, reactive oxygen species, and reorganization of the actin cytoskeleton. Activation of immune/inflammatory pathways and non-coding RNAs are also emerging as important regulators of vascular function. Vascular smooth muscle cell [Ca^2+^]_i_ not only determines the contractile state but also influences activity of many calcium-dependent transcription factors and proteins thereby impacting the cellular phenotype and function. Perturbations in vascular smooth muscle cell signalling and altered function influence vascular reactivity and tone, important determinants of vascular resistance and blood pressure. Here, we discuss mechanisms regulating vascular reactivity and contraction in physiological and pathophysiological conditions and highlight some new advances in the field, focusing specifically on hypertension.

## 1. Introduction

Hypertension is associated with vascular changes characterized by endothelial dysfunction, increased vascular contraction, and arterial remodelling.^1–3^ Vascular smooth muscle cells, which constitute the bulk of the vascular wall, are critically involved in these processes through their highly plastic and dynamic features and ability to undergo phenotypic differentiation.^1–3^ Pro-hypertensive stimuli, such as activation of the renin-angiotensin-aldosterone system (RAAS), oxidative stress, activation of the sympathetic nervous system, haemodynamic changes, and mechanical forces stimulate vascular smooth muscle cell signalling, which promotes vasoconstriction, vascular hypertrophy, fibrosis, inflammation, and calcification, processes that underlie vascular functional, structural, and mechanical changes in hypertension.^4^^,^^5^

The arterial system comprises large conduit vessels, medium sized arteries, small arteries, arterioles, and capillaries. Small resistance arteries (lumen diameter < 300 μm) are responsible for regional distribution of vascular tone and blood flow and play an important role in the regulation of blood pressure, through effects on vascular resistance.^6^ Three fundamental parameters determine resistance to blood flow including vessel diameter (radius), arterial length, and blood viscosity. Of these, vessel diameter is the most important because it can change rapidly due to contraction and dilation of vascular smooth muscle.^6^ Based on Poiseuille’s Law, vessel resistance is inversely proportional to the radius (lumen diameter) to the fourth power (*r*^4^). Therefore, small changes in lumen diameter have major impact on vascular resistance.^7^ The lumen diameter of resistance arteries is a function of vasomotor tone (vasoconstriction/vasodilation) and the structural and mechanical properties of the vessel. Vasomotor control underlies acute rapid adaptation of vessel diameter due mainly to vasoconstriction exerted by the active contraction of vascular smooth muscle cells, while changes in vascular structure represent a dynamic process in response to chronic haemodynamic changes.^8^ Initially structural changes are adaptive but in chronic pathological conditions become maladaptive leading to vascular remodelling and rigid, stiff, and poorly compliant vessels, typically observed in chronic hypertension.^9^^,^^10^ Endothelial stiffening decreases nitric oxide generation leading to smooth muscle cell contraction and vasoconstriction and may precede hypertension. Arterial stiffness is strongly associated with high blood pressure and is an independent predictor of cardiovascular disease.^11^^,^^12^

At the molecular and cellular levels vascular hyperreactivity, remodelling, and stiffening involve changes in cytoskeletal organization, cell-to-cell connections, cell growth, calcification, inflammation, and rearrangement of vascular smooth muscle cells.^13^^,^^14^ At the extracellular level, remodelling is influenced by fibrosis, changes in matrix protein composition and reorganization of proteoglycans, collagens, and fibronectin, which provide tensile strength, and elastin, responsible for vascular elasticity. Activation of adipocytes in perivascular adipose tissue, secrete vasoactive adipokines that also influence vascular reactivity and contractility.^15^^,^^16^

Acute regulation of vascular diameter and consequently vascular resistance depends on the activation status of the contractile machinery involving actin: myosin interaction in vascular smooth muscle cells.^17^ Changes in [Ca^2+^]_i_, ion fluxes, and membrane potential lead to calcium–calmodulin-mediated phosphorylation of the regulatory myosin light chains (MLCs) and actin–myosin cross-bridge cycling with consequent rapid vasoconstriction.^18^ Calcium-independent mechanisms associated with altered calcium sensitization and actin filament remodelling and increased bioavailability of reactive oxygen species (ROS) (oxidative stress), also modulate vascular contraction.^19^

In the present review, we discuss mechanisms regulating vascular reactivity and contraction in physiological and pathophysiological conditions, with a particular focus on hypertension. The role of vascular smooth muscle function in vascular remodelling, inflammation, and calcification and the importance of other vascular cell types in vascular health and disease are discussed elsewhere in the current issue of the journal.

## 2. The vascular media

The arterial wall is composed of three anatomical layers, a single layer of endothelial cells, the vascular media comprising multiple layers of vascular smooth muscle cells, and the adventitia, containing fibroblasts, adipocytes, connective tissue, and extracellular matrix.^20–23^ Endothelial cells secrete vasoactive agents and ROS that modulate the vessel diameter by influencing vascular smooth muscle cell function.^20^ The muscular media of vessels is innervated by the autonomic nervous system and its contractile state is regulated by hormones, vasoactive peptides, and ROS. Vascular smooth muscle cells possess a complex cytoskeletal skeleton, structural, and functional contractile proteins and associated regulatory molecules. Individual vascular smooth muscle cells connect to neighbouring cells through gap junctions, such as connexins, which control the synchronization in ion concentration and membrane potential between neighbouring cells.^22^^,^^23^

Although smooth muscle contraction can be tonic (sustained) or phasic, tonic contraction is essential for maintenance of vascular tone and regulation of blood flow. In resistance arteries vessels contract in response to increasing pressure in the physiological range (70–100 mmHg). With increased blood pressure, this myogenic response is impaired and basal vascular tone and contractility are increased.^24^ Clinically, non-invasive approaches (vascular ultrasound, pulse wave analysis, and peripheral arterial tone) to study endothelial function and vascular tone in humans have demonstrated that patients with hypertension exhibit impaired endothelium-dependent vasodilation, enhanced vascular reactivity, and increased contractility.^25^

## 3. The plastic nature of vascular smooth muscle cells

Vascular smooth muscle cells are specialized cells that are highly plastic and multifunctional. Physiologically vascular smooth muscle cells are quiescent and exhibit low levels of growth. Normally, they express genes and proteins important for contraction/dilation, which allows them to control systemic and local pressure through the regulation of vascular tone.^26^ However, under stressed or pathological conditions, highly differentiated contractile cells re-enter the cell cycle and become dedifferentiated, assuming a proliferative/migratory phenotype. Although differentiated cells express specific contractile and cytoskeletal proteins [e.g. smooth muscle cell α actin (α-SMA), smooth muscle myosin heavy chain (SM-MHC), calponin, caldesmon, and sm22-α], dedifferentiated cells express low levels of contractile markers and high levels of signalling molecules associated with cell growth, migration, fibrosis, and inflammation [e.g. cell cycle regulators (cyclins), mitogen-activated protein kinases (MAPKs), pro-inflammatory transcription factors, and matrix metalloproteinases].^27^

### 3.1 Vascular smooth muscle dedifferentiation in hypertension

In hypertension and other pathological conditions associated with vascular injury, the phenotypic switch contributes to vascular dysfunction and arterial remodelling. In the early stages of hypertension and during vascular repair, the cell cycle is tightly regulated contributing to controlled vascular smooth muscle cell proliferation important in the ‘adaptive’ phase of remodelling. However when the cell cycle is unchecked and proliferation is uncontrolled, dedifferentiated smooth muscle cells accumulate in the vascular wall leading to media thickening, neointimal hyperplasia, and vascular stiffness, features found in hypertension, atherosclerosis, and pulmonary artery hypertension.^28^ Studies using cell-tracking approaches in *in vivo* models, demonstrated that >80% of vascular smooth muscle cells in sites of arterial injury or vascular remodelling exhibit features of dedifferentiation.^29^

### 3.2 Molecular mechansims of vascular smooth muscle cell dedifferentiation in hypertension

Molecular mechanisms underlying the cellular phenotypic switch in hypertension are complex and multifactorial. Vasoactive stimuli [Angiotensin II (Ang II), norepinephrine, and endothelin-1 (ET-1)], growth factors [Insulin-like growth factor 1 (IGF-1), epidermal growth factor (EGF), platelet-derived growth factor (PDGF)], mechanical forces (stretch), and physical factors (shear stress, pressure) are important.^30^^,^^31^ These processes induce changes in expression and function of genes that control cell membrane receptors, growth signalling pathways, extracellular matrix components, transcription factors, ion channels, and transporters, important in vascular hypertrophy in hypertension.^31–33^

### 3.3 The non-coding genome and dedifferentiation of vascular smooth muscle cells in hypertension

Recent evidence indicates that the phenotypic switch of smooth muscle cells involves the non-coding genome. Non-coding RNAs (ncRNAs), which are classified based on their size: small ncRNAs (<200 nucleotides) and long ncRNAs (lncRNA) (>200 nucleotides), regulate gene expression at multiple levels including transcription, RNA processing, and translation.^26^^,^^34^ Most ncRNAs do not have protein-coding abilities but they guide DNA synthesis or genome rearrangement and as such have major impact on the regulation of the genome.

Many classes of small ncRNAs have been identified, of which miRNAs are particularly important in the phenotypic regulation of smooth muscle cells. Mature miRNA derives from precursor miRNA through cleavage by the enzyme Dicer. Evidence supporting a pathophysiological role for miRNA in smooth muscle cell differentiation and proliferation derives from *in vivo* studies in conditional knockout mice, where smooth muscle cell-specific knockout of Dicer was associated with dilated, thin-walled arteries, reduced vascular smooth muscle cell proliferation, decreased expression of contractile genes, and decreased blood pressure.^34^^,^^35^ Similar features were observed in mice deficient in smooth muscle cell miR-143/145 cluster.^36^ Other miRNAs associated with vascular smooth muscle cell differentiation include miR-21, miR-22, miR-26a, miR-34a, miR-146a, and miR-221/222.^26^^,^^34–36^

Although the list of miRNAs involved in vascular smooth muscle cell differentiation is growing, there is a paucity of information on vascular cell lncRNAs. LncRNA regulate gene expression by stimulating or repressing gene transcription, translation, and signalling. They also regulate the structure and function of chromosomes. Multiple lncRNAs, including H19, ANRIL, lncRNA-p21, lncRNA-362, and GAS5, have been associated with vascular smooth muscle cell differentiation/proliferation and various vascular pathologies.^37^ Most lncRNA are widely expressed, however, smooth muscle and endothelial cell enriched migration/differentiation-associated lncRNA (SENCR) seems to be specifically expressed in smooth muscle cells and endothelial cells.^35^ Numerous Ang II-regulated lncRNAs have been identified in vascular remodelling in experimental hypertension.^38^ In particular, lncRNA-GAS5 (growth arrest-specific 5) has been identified as a regulator of hypertension-induced vascular remodelling, which when knocked down causes hypertension.^39^

## 4. Vascular contraction and hypertension

Dynamic changes in vascular diameter depend in large part on the contractile activation and inactivation (phosphorylation/dephosphorylation) of contractile proteins in vascular smooth muscle cells. The contractile machinery of vascular smooth muscle includes actin and myosin and a highly organized cytoskeleton.

### 4.1 A primer in vascular smooth muscle contraction: Ca^2**+**^-dependent mechanisms

**Figure 1:**
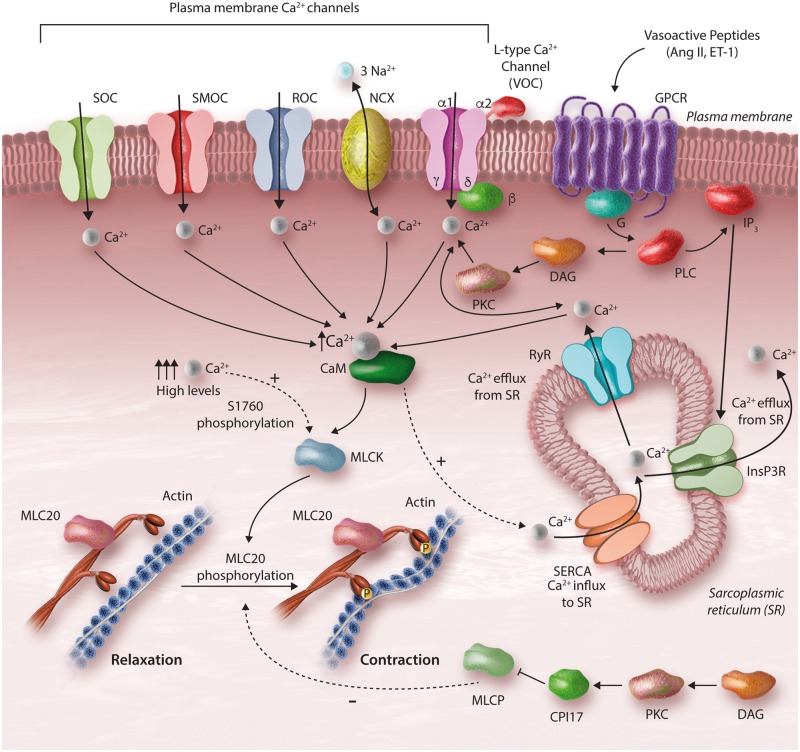
Calcium-dependent regulation of vascular smooth muscle cell (VSMC) contraction. Vasoconstrictors induce VSMC contraction by increasing the intracellular levels of Ca^2+^. Vasoactive peptides, such as Ang II, bind to G protein-coupled receptors (GPCRs) activating PLC leading to (i) production of IP_3_ and (ii) formation of DAG. IP_3_ binds to the IP receptor Ca^2+^ channel (InsP3R) and induces Ca^2+^ release from the sarcoplasmic reticulum (SR). DAG causes activation of PKC, which influences Ca^2+^ channels, such as store-operated Ca^2+^ channel (SOC), second messenger-operated Ca^2+^ channel (SMOC), receptor-operated Ca^2+^ channel (ROC), voltage-gated Ca^2+^ channel (VOC), and the Na^+^–Ca^2+^ exchanger (NCX). PKC also stimulates activity of the ryanodine Ca^2+^ channel (RyR) inducing release of Ca^2+^ from the SR. MLCP activity is reduced by CPI-17 phosphorylation. Ca^2+^ binds to calmodulin and activates the MLCK, leading to MLC20 phosphorylation at Ser19, actin polymerization, and vascular contraction.

The key event in vascular smooth muscle excitation-contraction coupling is an increase in [Ca^2+^]_i_ in response to mechanical, humoral, or neural stimuli^40^^,^^41^ (*Figure 1*). [Ca^2+^]_i_ and calcium signalling control the key functions of vascular smooth muscle cells and are finely tuned by plasma membrane calcium-permeable channels, exchangers, and transporters and by intracellular sources, including the sarcoplasmic reticulum, mitochondria, and calcium-binding proteins. The extracellular concentration of calcium is 2–4 mM with a basal [Ca^2+^]_i_ of 90–110 nM.^40^^–^^42^ In hypertension, these processes are altered leading to increased [Ca^2+^]_i_ and a hypercontractile state and vascular remodelling.

Stimulation of vascular smooth muscle cells by pro-hypertensive factors such as neurohumoral stimuli (acetylcholine, norepinephrine) and vasoactive peptides (Ang II, ET-1), induces activation of receptors coupled to Phospholipase C (PLC), leading to generation of the second messengers inositol trisphosphate (IP_3_) and diacylglycerol (DAG).^31^^,^^40–42^ Circulating non-cellular factors such as cytokines, diffusible ROS (nitric oxide and hydrogen peroxide), and miRNAs and cellular-derived factors such as microparticles and endothelial progenitor cells also stimulate membrane receptors or cross the plasma membrane to regulate pathways that control [Ca^2+^]_i_.^43–45^ Moreover, endothelium-secreted vasoconstrictors regulate vascular contraction. Endothelial-derived ET-1 and prostanoids activate vascular smooth muscle cell receptors leading to activation of pro-contractile signalling.^46^

PLC-induced IP_3_ production stimulates intracellular calcium release from the sarcoplasmic reticulum and DAG causes activation of protein kinase C (PKC). In addition, numerous calcium channels, such as voltage-operated (VOC), receptor-operated channels (ROC), store-operated channels (SOC), transient receptor potential cation (TRP) channels and Ca^2+^-permeable non-selective cation channels are activated, promoting calcium influx and increased [Ca^2+^]_i_.^47–51^ Calcium diffuses to the contractile machinery and binds to calmodulin. The calcium–calmodulin complex induces a conformational change in MLC kinase (MLCK) converting it from an inactive to an active state.^40–43^ Activated MLCK induces phosphorylation of MLC20, stimulates myosin–actin interaction, which generates force and shortening and consequent vascular contraction.

### 4.2 Calcium-dependent mechanisms of vascular smooth muscle cell contraction in hypertension

In hypertension, many of the mechanisms regulating intracellular calcium homeostasis are perturbed, with experimental models and hypertensive patients demonstrating abnormal vascular calcium handling and high [Ca^2+^]_i_. Increased calcium influx, augmented sarcoplasmic reticular calcium release and decreased sarcoplasmic reticular calcium reuptake, activation of the PLC-DAG-IP_3_ pathway, increased calcium signalling, vascular hyperreactivity, and exaggerated contractile responses to vasoactive agonists have been demonstrated in genetic [spontaneously hypertensive rats (SHRs), stroke-prone SHR], experimental (deoxycorticosterone acetate (DOCA)-salt, Ang II-infused, L-NAME-induced), and human hypertension.^47–54^ Processes triggering these events in hypertension likely involve activation of the sympathetic nervous system and up-regulation of the RAAS.

The importance of calcium channels in abnormal calcium handling in hypertension is evidenced by the effective antihypertensive actions of L-type calcium channel blockers, which target L-type CaV1.2 calcium channels.^55^^,^^56^ In experimental and clinical hypertension, activation of L-type VOCs is increased and sensitivity to calcium channel blockers is greater in hypertensive compared with normotensive individuals.^55^^,^^56^ Processes underlying this include increased expression and phosphorylation of subunits of L-type calcium channels.^55^ Other mechanisms involving regulatory factors have also been identified. In particular, recent studies demonstrated that the splicing factor Rbfox2 plays a regulatory role in Ca_V_1.2 calcium channels and calcium influx in vascular smooth muscle cell contraction. In hypertension, aberrant splicing by dysregulated Rbfox2 induces enhanced activity of Ca_V_1.2 calcium channels leading to increased vascular myogenic tone.^57^

ROCs, TRP channels (TRPC3, TRPC6, and TRPC7), and the Na^+^/Ca^2+^ exchanger (NCX) have also been shown to play a role in altered calcium handling and vascular dysfunction in hypertension.^50^ Moreover, processes that stimulate store-operated calcium entry (SOCE) may be important. SOCE, a mechanism whereby reduced sarcoplasmic reticular calcium stores stimulate calcium influx, is influenced by stromal interaction molecule 1 (STIM1) and Orai1 (pore subunit of calcium release-activated calcium channels). In hypertension, increased expression of vascular STIM1/Orai1 is associated with augmented aortic contraction, especially in males.^50^^,^^58^

In hypertension, vascular smooth muscle calcium homeostasis is also modulated by activation of signalling pathway not typically associated with contration, such as MAPKs, tyrosine kinases, transcription factors, and nicotinamide adenine dinucleotide phosphate (NADPH) oxidases (Noxs).^59–63^ We showed that c-Src is a point of cross-talk between calcium- and oxidation/reduction (redox)-signalling in vascular cells and that in human hypertension, up-regulation of this system contributes to increased vascular [Ca^2+^]_i_ and contraction.^64^ Redox-regulated calcium-sensitive transcription factors, including serum response factor (SRF), nuclear factor of activated T-cells (NFAT), and cyclic adenosine monophosphate (cAMP) responsive element-binding protein, promote expression of genes encoding contractile proteins, further influencing vascular contractility in hypertension.^50^

Involvement of immune and inflammatory systems has also been shown to influence vascular calcium homeostasis in hypertension and target organ damage in hypertension. Activated immune cells migrate into target organs, including the vasculature, and factors released by these cells, including ROS, metalloproteinases, cytokines, and chemokines promote dysfunction and cause damage.^65^ In vessels, these factors enhance constriction, remodelling, and rarefaction. Vascular toll-like receptors (TLRs) and damage-associated molecular patterns (DAMPs) have also been shown to influence vascular reactivity in hypertension. TLR4 is up-regulated in resistance arteries of SHR and contributes to hypercontractile responses through DAMP and cyclooxygenase-dependent pathways.^66^ In hypertension, increased DAMP-TLR activation may influence vascular reactivity as well as elicit a vascular inflammatory response, also important in the pathophysiology of hypertension. Involvement of immune cells in the perivascular adventitial and adipose tissue has also been implicated in hypertension-associated vascular dysfunction.^67^

### 4.3 Calcium-independent mechanisms of vascular smooth muscle cell contraction in hypertension: calcium sensitization

**Figure 2:**
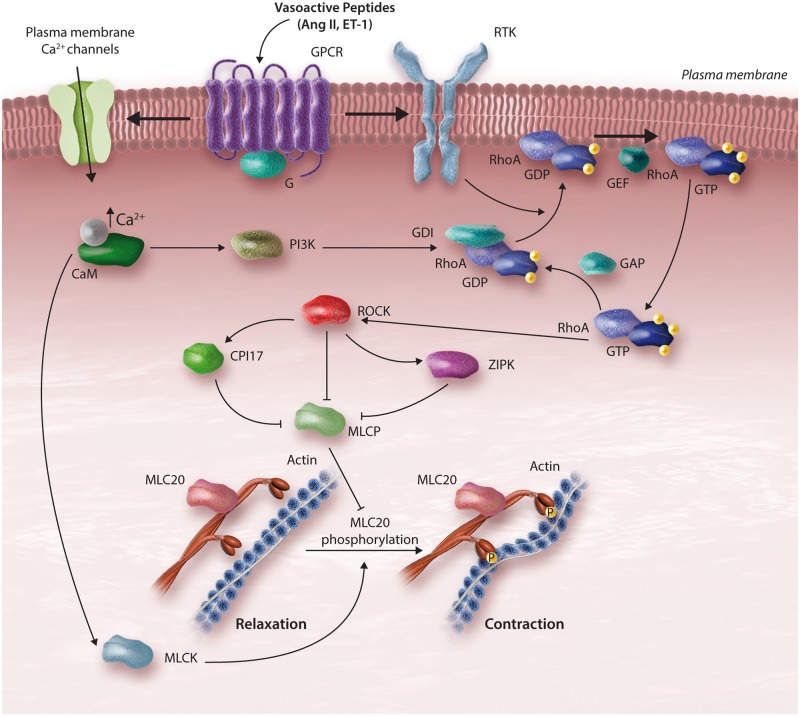
ROCK-induced contraction of VSMCs. Vasoactive agents bind to their respective GPCRs leading to release of RhoA from a guanine nucleotide dissociation inhibitor (GDI). RhoA translocates to the membrane. Mechanisms involving RhoA activation also involve transactivation of receptor tyrosine kinases (RTKs). GEFs promote exchange of Guanosine diphosphate (GDP) to Guanosine triphosphate (GTP), activating the RhoA-ROCK pathway. Activated ROCK renders MLCP inactive by phosphorylation of CPI-17 and/or zipper-interacting protein (ZIPK), facilitating MLC20 phosphorylation and vascular contraction. Increased Ca^2+^ may directly activate ROCK through phosphatidylinositol-4, 5-bisphosphate 3-kinase (PI3K)-dependent pathways.

In addition to calcium-dependent mechanisms, calcium-independent processes regulate vascular smooth muscle contraction, by influencing the sensitivity of MLC to calcium. The calcium sensitization process maintains force generation following dissipation of the initial calcium signal. Two major signalling pathways have been implicated in calcium sensitization, including the DAG-PLC-PKC pathway and the RhoA-Rho kinase (ROCK) pathway^67^^,^^68^ (*Figure 2*). Other kinases also play a role including integrin-linked kinase (ILK), p21-activated protein kinase and zipper-interacting protein kinase (ZIPK).^69^^,^^70^ The calcium sensitization mechanism regulates the phosphorylation state of MLC20 independently of calcium–calmodulin–MLCK signalling.

Hydrolysis of phosphatidylinositol 4, 5-bisphosphate (PIP2) by PLC produces IP_3_ and DAG. IP_3_ stimulates release of calcium from the sarcoplasmic reticulum, while DAG acts as a second messenger inducing activation of PKC and C-kinase potentiated protein Phosphatase 1 inhibitor, molecular mass 17 kDa (CPI-17). PKCs are classified in three major groups; conventional or classic PKCs, novel PKCs, and atypical PKCs. Vascular smooth muscle cells variably express multiple isoforms, although the classic isoforms, α, β, and γ appear to be most abundant. Activated PKC induces phosphorylation of downstream targets including ion channels, transporters, and nuclear protein.^71^ It also phosphorylates CPI-17, a smooth muscle-specific inhibitor of MLC phosphatase (MLCP), which binds to its catalytic domain, inhibiting phosphatase activity, facilitating persistent contraction. α-PKC also induces phosphorylation of actin-binding proteins, such as calponin and calmodulin, which promote actin–myosin interaction and vascular smooth muscle cell contraction.^72^

Increased PKC activity, associated with vascular hypercontractility, has been demonstrated in experimental and clinical hypertension. Agonist-stimulated vasoconstriction is more potently inhibited by PKC inhibitors in aorta from SHR vs. control Wistar Kyoto rats.^73^ Moreover, hindlimb perfusion of a phorbol ester that activates PKC, caused prolonged vasoconstriction, and increased perfusion pressure in SHR compared with normotensive WKY, effects that were blocked by PKC inhibitors.^74^ Human vascular smooth muscle cells from hypertensive patients exhibit increased Ang II-induced proliferation and contraction through processes that involve Phospholipase D and PKC.^75^ Several PKC inhibitors have been developed, including non-specific staurosporine and chelerythrine, which reduce contraction in hypertension. Isoform-specific PKC inhibitors, such as ruboxistaurin have been tested in clinical trials for diabetic retinopathy^76^ and may have potential in the treatment of other vascular diseases.

The other system that sensitizes myosin to calcium involves ROCKs (ROCK1, ROCK2), which are serine/threonine kinases and downstream effectors of the small GTPase RhoA.^77^^,^^78^ RhoA, a member of the Rho family of small GTPase-binding proteins, is abundantly expressed in vascular smooth muscle cells and is rapidly activated by vasoconstrictors, such as Ang II. ROCK influences calcium sensitization through two main mechanisms. First, it stimulates phosphorylation of myosin phosphatase target subunit 1 (MYPT1) at T695 or T853. Secondly, it phosphorylates ZIPK (also known as DAPK3), which stimulates phosphorylation of MYPT1 at T696 and T18/S19.^79^^,^^80^ MYPT1 phosphorylation in turn interferes with binding of MLCP to MLC, and accordingly reduces phosphatase activity leading to sustained contraction. In addition to MYPT1, ROCK phosphorylates CPI-17.^81^ Hyperactivation of the RhoA-ROCK leads to decreased MLCP activation and consequent sustained vasoconstriction and blood pressure elevation. ROCK-dependent calcium sensitization is particularly important in the acute phase of vasoconstriction, as evidenced by studies demonstrating that blockade of this pathway reduces the initial vasoconstrictor action of mechanical, humoral, and neural stimuli.^82^

Experimental models of hypertension exhibit increased vascular RhoA/ROCK activation,^76^^,^^83–86^ processes that are augmented with high salt diet.^83^ A number of mechanisms have been implicated in ROCK hyperactivation in hypertension, including dysregulation of Rho guanine nucleotide exchange factors (Rho-GEFs).^84^ In experimental hypertension, activation of ROCK signalling generates a negative feedback loop on the expression of vascular RhoA-GEFs, which influences ROCK-dependent contraction or remodelling in vessels under pathophysiological conditions, including hypertension.^85–88^ In clinical studies, mononuclear cell p63RhoGEF gene and protein expression and MYPT1 phosphorylation were increased in patients with essential hypertension.^88^ ROCK2 also plays an important role in Ang II-induced hypertension and cardiac hypertrophy, through processes that involve formin Homology 2 domain containing 3 (FHOD3), crucial in regulating myofibrillogenesis in cardiomyocytes.^87^ In human vascular smooth muscle cells, Ang II activates ROCK-mediated contractile signalling through the RhoA exchange factor Arhgef1.^88^ In addition to modulating vascular contraction in hypertension, ROCK activation is associated with increased vascular stiffness through processes that increase SRF/myocardin signalling.^89^

Pharmacological inhibition of ROCK with fasudil or Y27632 suppresses acute pressor responses of Ang II but does not reduce blood pressure chronically, supporting the role of RhoA/ROCK in acute vasoconstriction, rather than in mechanisms associated with adaptive vascular remodelling in hypertension that occur chronically with Ang II infusion.^90–93^ Clinical studies have demonstrated beneficial effects of ROCK inhibitors. Fasudil has been used clinically and prevents vasospasm associated with subarachnoid haemorrhage, acute ischaemic stroke, angina, coronary artery spasm, atherosclerosis, and in the regulation of vascular tone in hypertensive renal transplant recipients.^91–96^ Increasing evidence indicates a pathophysiological role for ROCK in pulmonary arterial hypertension, with clinical studies suggesting ROCK inhibitors as potential therapeutic agents.^96^ Use of ROCK inhibitors in human essential hypertension is not yet approved but may be an interesting strategy.

## 5. Reversal of vascular smooth muscle contraction—importance of vascular relaxation

Vascular smooth muscle relaxation occurs as a result of decreased [Ca^2+^]_i_ due to inactivation of L-type calcium channels with reduced calcium influx, increased plasmalemmal Ca^2+^ATPase and activation of the sodium–calcium exchanger with increased calcium efflux and activation of sarcoplasmic/endoplasmic reticulum Ca^2+^ATPase (SERCA), which stimulates reuptake of calcium into the sarcoplasmic reticulum. As calcium dissociates from calmodulin, MLCK becomes inactivated and MLC20 is dephosphorylated by the serine/threonine phosphatase MLCP.^97^^,^^98^ The overall magnitude of MLC20 phosphorylation and resultant vascular smooth muscle contraction are determined by the relative activities of MLCK and MLCP. Another pathway promoting vascular relaxation involves endothelial-derived nitric oxide that regulates cyclic guanosine monophosphate and cAMP, which signal through protein kinase G (PKG) and protein kinase A (PKA) to increase MLCP activity and decrease calcium sensitivity of the contractile machinery.^99^^,^^100^

Impaired vasorelaxation, with sustained contraction and altered MLCP activity have been demonstrated in human and experimental hypertension.^101^ In smooth muscle from mice with specific knockout of the MLCP regulatory subunit MYPT1, phosphorylation of MLC and contractile force in isolated mesenteric arteries were enhanced, indicating an important role for MLCP and its regulatory subunit MYPT1 in vascular contraction and development of hypertension.^102^

## 6. The actin cytoskeleton and contraction

In addition to the calcium-dependent and -independent modulation of MLC20 phosphorylation and actin–myosin cross-bridge formation, reorganization of actin filaments, intermediate filaments, and microtubules play an important role in vascular contraction. Increased polymerization of actin, tyrosine phosphorylation of paxillin, activation of small GTP-binding proteins, and conformational changes in focal adhesion sites result in stiffening and reorganization of the cytoskeleton in vascular smooth muscle cells.^103^ This dynamic rearrangement of the actin cytoskeleton is key in maintaining vascular tone and plasticity, especially important in the regulation of vascular diameter related to pressure-dependent myogenic tone in hypertension. Cytoskeletal remodelling occurs over time, with actin polymerization increasing as vasoconstriction persists.^104^ This involves cellular reorganization and formation of new intercellular adhesions through integrins and focal adhesion molecules.^105^^,^^106^ Prolonged vasoconstriction and associated actin polymerization participate in the initial phases of vascular remodelling in hypertension, processes that involve ROCK and ROS.^107^ Vascular smooth muscle cell length adaptation, with reorganization of the actin cytoskeleton also contributes to small artery eutrophic remodelling, typically observed in essential hypertension.^108^

## 7. Reactive oxygen species, calcium, and contraction in hypertension

Similar to intracellular calcium, ROS are now considered important second messenger molecules in vascular smooth muscle cells in that (i) they are generated within cells in a regulated manner, (ii) they act on downstream signalling molecules, and (iii) their effects on protein targets reversibly influence cellular function.^109^ ROS (superoxide anion and hydrogen peroxide) and reactive nitrogen species (nitric oxide and peroxynitrite) are biologically important O_2_ derivatives that play a role in vascular (patho)biology through their redox potential. The major enzymatic source of ROS in endothelial and vascular smooth muscle cells is Nox. Seven Nox isoforms have been identified, of which Nox1, 2, 4, and 5 are expressed and functionally active in human vascular smooth muscle cells.^110–112^ Noxs are activated by vasoactive agents and activation is increased in hypertension.^113^^,^^114^ Superoxide is converted to the more stable hydrogen peroxide by superoxide dismutase (SOD). Hydrogen peroxide is further reduced to water and oxygen by catalase and peroxidases. In endothelial cells, superoxide reacts with nitric oxide producing peroxynitrite. An imbalance in redox state where pro-oxidants overwhelm antioxidant capacity leads to oxidative stress, which in turn causes vascular inflammation, fibrosis and arterial remodelling in hypertension.^115^

**Figure 3:**
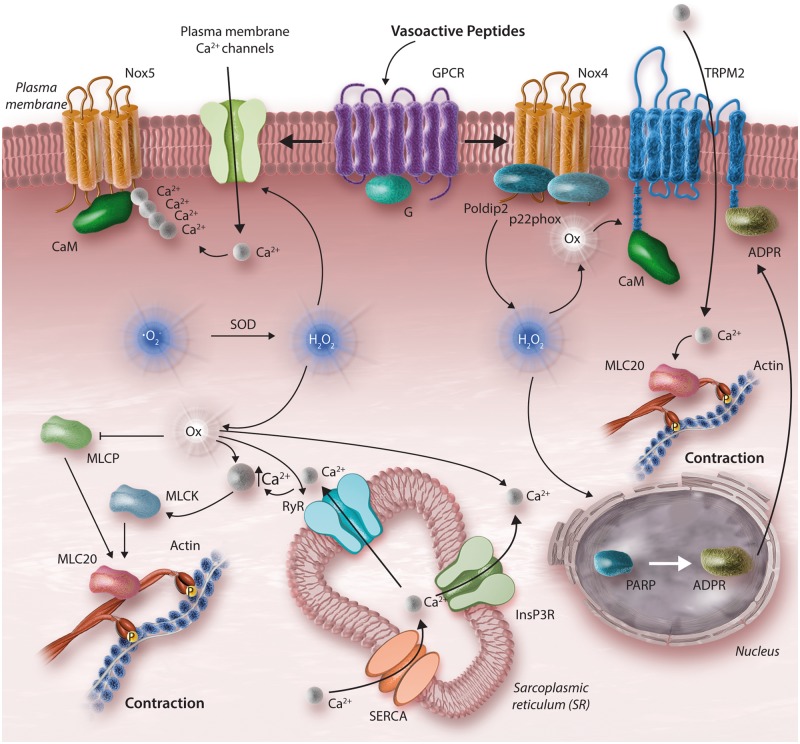
VSMC contraction and oxidative stress. Nox-derived ROS influence cellular Ca^2+^ homeostasis and pro-contractile signalling. Calcium also influences Nox-derived ROS generation, through Nox5, a Ca^2+^-sensitive Nox isoform that produces $O_{2}^{\text{−}}$. Nox4 activation leads to production of H_2_O_2_. High levels of ROS, cause oxidative modifications of Ca^2+^ channels, ryanodine receptors (RyR), actin, actin-binding proteins as well as TRP cation channel, subfamily M, Member 2 (TRPM2). TRPM2, a redox-sensitive Ca^2+^ channel indirectly activated by H_2_O_2_ through poly (ADP-ribose) polymerase (PARP) activation in the nucleus and consequent Adenosine diphosphate-ribose (ADPR) production. Once released to the cytosol, ADPR binds and activates TRPM2, stimulating Ca^2+^ influx. Dashed line indicates that reduced MLCP activity results in icreased MLC20 activation.

Vascular ROS effects are mediated through redox-sensitive signalling pathways. ROS regulate protein kinases, phosphatases, MAPKs, and transcription factors and are important modulators of [Ca^2+^]_i_, ROCK, and the contractile machinery (*Figure 3*). ROS increase vascular [Ca^2+^]_i_ by stimulating IP_3_-mediated calcium mobilization, by increasing cytosolic calcium accumulation through SERCA inhibition, and by stimulating calcium channels.^116^^,^^117^ Increased Nox-derived ROS generation enhances calcium signalling, up-regulates ROCK and modulates the actin cytoskeleton, thereby promoting vascular contraction and increasing vascular tone.

Superoxide causes vasoconstriction while hydrogen peroxide induces both vasodilation and vasoconstriction, depending on the vascular bed and the Nox isoform involved. In cerebral arteries, coronary arteries, and pulmonary vessels, hydrogen peroxide is a potent vasodilator,^118^^,^^119^ whereas in peripheral arteries and aorta it causes vasoconstriction.^120^^,^^121^ Hydrogen peroxide acts as an endothelium-derived hyperpolarizing factor and induces vasodilation through PKG1α-mediated pathways^122^^,^^123^ and inhibition of intracellular calcium mobilization, while ROS mediates vasoconstriction by increasing [Ca^2+^]_i_.^52^

## 8. Oxidative stress, protein oxidation, and vascular contraction

ROS influence pro-contractile signalling through post-translational oxidative modification of proteins. Cysteine and methionine residues on proteins are highly redox-sensitive and undergo oxidative modification when ROS production is increased, such as in hypertension.^124^ Under physiological conditions, protein oxidation is usually reversible, while in pathological conditions associated with oxidative stress, protein oxidation may be irreversible resulting in oxidative damage of proteins and cell death. Many proteins that regulate vascular smooth muscle cell calcium homeostasis, including calcium channels, SERCA, TRP channels, and ROCK, undergo post-translational oxidative modifications.^125–127^ In addition, actin and actin-binding proteins, myosin and cofilin, are directly oxidized by ROS.^128^^,^^129^ Accordingly, increased vascular Nox-derived ROS generation in hypertension causes an increase in [Ca^2+^]_i_ and cytoskeletal rearrangement leading to altered vascular reactivity and enhanced contraction.

## 9. Conclusions

Vascular smooth muscle cells are highly differentiated and normally maintain a contractile phenotype. Vascular contraction/relaxation is regulated by many processes that are both calcium-dependent and -independent and involve calcium channels and signalling pathways such as IP_3_-PKC-DAG and ROCK. In hypertension, these processes are dysregulated, and signalling pathways not typically associated with contraction, such as MAPKs, tyrosine kinases, and transcription factors are activated. These phenomena lead to a hypercontractile state and dedifferentiation of vascular smooth muscle cells to a proliferative/migratory phenotype with consequent vascular remodelling. Emerging evidence also implicates a role for the immune/inflammatory system and the non-coding genome in vascular dysfunction in hypertension. Unravelling the complex interactions between traditional pro-contractile calcium-regulated signalling pathways and non-traditional contractile mechanisms will provide better insights into processes underlying the vascular phenotype in hypertension.

**Conflict of interest**: none declared.

## Funding

R.M.T. was supported through a British Heart Foundation Chair award (CH/4/29762). A.C.M. was supported through a University of Glasgow Walton fellowship. R.A.-L. was supported through a BHF Award of Excellence (RE/13/5/30177).
